# Alpha‐tACS Improves Executive Function and Alpha Power Spectral Density in Patients With Post‐Stroke Cognitive Impairment

**DOI:** 10.1002/cns.70963

**Published:** 2026-06-17

**Authors:** Weicheng Kong, Jian Song, Yanyan Li, Yuqing Zhao, Xinyang Wang, Haoran Shi, Jiayu Cai, Wei Wei, Xiehua Xue

**Affiliations:** ^1^ The Affiliated Rehabilitation Hospital Fujian University of Traditional Chinese Medicine Fuzhou China; ^2^ College of Rehabilitation Medicine Fujian University of Traditional Chinese Medicine Fuzhou China; ^3^ Fujian Provincial Rehabilitation Industrial Institution, Fujian Provincial Key Laboratory of Rehabilitation Technology Fujian Key Laboratory of Cognitive Rehabilitation Fuzhou China

**Keywords:** alpha‐frequency, electroencephalography, executive impairment, stroke, transcranial alternating current stimulation

## Abstract

**Background:**

This study aims to evaluate the efficacy of alpha‐frequency transcranial alternating current stimulation (alpha‐tACS) in treating post‐stroke cognitive impairment (PSCI) and its effects on electroencephalographic activity.

**Methods:**

Forty PSCI patients were randomly assigned to receive either active alpha‐tACS (10 Hz, 2 mA over bilateral DLPFC) or sham stimulation for 4 weeks, combined with standard therapy. Cognitive function was assessed using MoCA and TMT‐A/B, complemented by resting‐state EEG. ROC analysis was employed to evaluate alpha power spectral density (PSD).

**Results:**

Following the 4‐week intervention, the alpha‐tACS group demonstrated significantly greater improvements in MoCA scores and TMT‐A completion times compared to the sham group (*p* < 0.05). Alpha power spectral density (PSD) increases were significantly more pronounced in the alpha‐tACS group across all electrodes. Additionally, this group exhibited significantly greater reductions in whole‐brain DAR and DTABR values (*p*_FDR_ < 0.05). Correlation analysis revealed significant positive associations between TMT‐A improvements and alpha PSD changes at multiple electrodes (F3, F4, F7, F8, C3, C4, P4, T4, O2, FZ, CZ, PZ; *p*_FDR_ < 0.05). Regression analysis identified P3 electrode PSD difference as a significant predictor (*p* = 0.01), with ROC analysis showing AUC values of 0.685 (T1 comparison) and 0.797 (T1–T0 comparison).

**Conclusion:**

Alpha‐tACS effectively enhances alpha oscillations and improves executive functions in PSCI patients, supporting its potential as a promising neuromodulatory intervention for cognitive rehabilitation.

## Graphical abstract

We applied alpha‐frequency transcranial alternating current stimulation (alpha‐tACS) to treat post‐stroke cognitive impairment. A 4‐week intervention significantly improved cognitive scores and executive function while enhancing alpha oscillations across cortical networks.

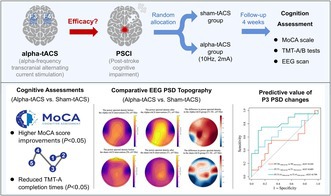

## 1 Introduction

Post‐stroke cognitive impairment (PSCI) is a clinical syndrome defined as any cognitive impairment that occurs after stroke events [1]. PSCI affects approximately 38% of stroke survivors [2] and places a significant burden on these individuals [3]. Therefore, developing effective interventions to improve cognitive function in stroke survivors is a key clinical priority.

Non‐invasive neuromodulation techniques have recently emerged as promising tools for treating cognitive disorders. Transcranial alternating current stimulation (tACS) is one such novel neuromodulation technique that is increasingly being applied to treat cognitive disorders [4]. tACS can be delivered at various stimulation frequencies, including the delta (1–4 Hz), theta (4–8 Hz), alpha (8–13 Hz), and beta (13–30 Hz) bands, and its clinical effect varies depending on the frequency selected.

Stroke patients often exhibit reduced alpha band activity in the affected hemisphere [5]. Alpha oscillations are critically involved in the execution of internally driven tasks, particularly in the visual domain [6, 7, 8]. As such, alpha activity serves as an important neurophysiological marker of cognitive function. Notably, PSCI patients frequently experience significant impairments in attention, working memory, and executive function [1]. Recent studies have suggested that alpha‐tACS can noninvasively induce lasting modulation of endogenous alpha oscillations [9, 10]. As a result, it is being investigated as a potential technique to ameliorate various post‐stroke symptoms [11]. Both 10 and 20 Hz tACS were found to facilitate motor sequence learning during a serial reaction time task [12]. Alpha‐tACS has also shown significant potential for treating post‐stroke hemispatial neglect [13]. However, research on the use of 10 Hz alpha‐tACS for treating post‐stroke cognitive impairment is limited.

Although the underlying neurophysiological mechanisms of alpha‐tACS are not fully understood, elucidating how it influences cognition in stroke patients is crucial. This study aimed to investigate the effects of alpha‐tACS on patients with basal ganglia stroke. We used electroencephalography (EEG) power spectrum analysis to examine its modulatory effects on the dorsolateral prefrontal cortex (DLPFC).

## 2 Materials and Methods

### 2.1 Participants

Patients with PSCI secondary to basal ganglia stroke were recruited from the Affiliated Rehabilitation Hospital of Fujian University of Traditional Chinese Medicine between April 24, 2024, and March 30, 2025. Clinical and demographic data were collected, including medical history (e.g., hypertension or diabetes), age, education years, smoking status, and alcohol consumption. The study protocol was approved by the Ethics Committee of the Rehabilitation Hospital of Fujian University of Traditional Chinese Medicine (2024YJS‐003‐01) and was registered with the Chinese Clinical Trial Registry (ChiCTR2500095137).

Inclusion criteria were as follows: (1) Diagnosis of ischemic or hemorrhagic stroke in the basal ganglia region, confirmed by computed tomography (CT) or magnetic resonance imaging (MRI) and diagnosed by an experienced, certified physician. (2) Experienced their first‐ever stroke (ischemic or hemorrhagic) within the past 3–6 months. (3) Aged 45–80 years. (4) A Montreal Cognitive Assessment (MoCA) score of ≤ 24 [14]. (5) Absence of metallic implants or cardiac pacemakers. (6) Ability to understand and cooperate with the required assessments. (7) Provided written informed consent, either personally or from a legal guardian or immediate family member.

Exclusion criteria were as follows: (1) Cognitive impairment due to other conditions such as brain tumors, Alzheimer's disease, hypothyroidism, or traumatic brain injury. (2) A history of lacunar infarction with residual neurological deficits. (3) A personal or family history of epilepsy or psychiatric disorders. (4) Consciousness disturbances, or severe impairments in vision, hearing, or speech. (5) Fever, electrolyte imbalance, or unstable vital signs. (6) Severe dysfunction of major organs, including heart, lungs, liver, or kidneys. (7) Contraindications to tACS such as local skin lesions or infections, cranial defects, or hypersensitivity at the stimulation site.

### 2.2 Experimental Design

The randomization sequence was computer‐generated and concealed in sequentially numbered, opaque envelopes by an independent statistician. Participants were assigned 1:1 to tACS or control groups. A double‐blind design was implemented through coded labeling (A/B system), with outcome assessors and statisticians blinded to group allocation. Unblinding occurred after database lock and completion of statistical analysis under independent supervision.

A total of 44 patients with PSCI were randomly assigned to either the alpha‐tACS group (*n* = 22) or the sham‐tACS group (*n* = 22). Four patients (two from each group) withdrew from the study during the intervention period (two were transferred to other hospitals and two were unable to continue the intervention), resulting in a final sample of 20 patients per group. Each participant received 20 sessions of either alpha‐tACS or sham‐tACS stimulation applied to the DLPFC.

**FIGURE 1:**
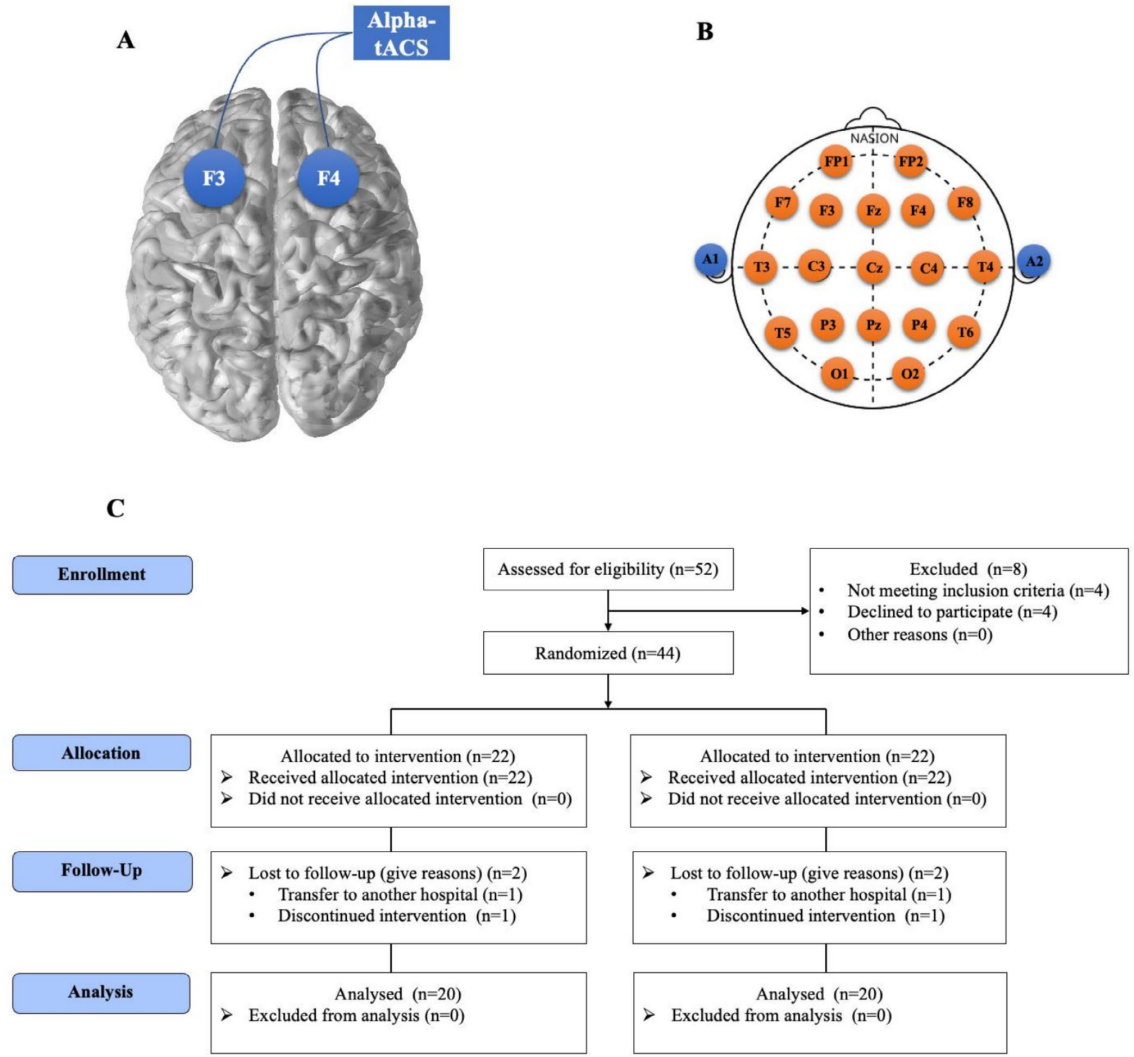
tACS stimulation point, EEG electrode and research flowchart. (A) The stimulation area of alpha‐tACS (the F3 and F4 electrodes in the EEG). (B) The 10–20 international standard 19‐channel electrode distribution map. (C) The research flowchart.

The interventions were administered once a day, 5 days a week (Monday–Friday), for four consecutive weeks. All enrolled participants received identical conventional pharmacotherapy and cognitive rehabilitation interventions throughout the study period. Cognitive function and EEG were collected pre‐ (T0) and post‐intervention (T1) in both groups, see Figure 1.

### 2.3 Sample Size Estimation

Sample size was calculated using GPower software. Given the limited published evidence on transcranial alternating current stimulation (tACS) for post‐stroke cognitive impairment (PSCI), and based on the premise that tACS exerts cognitive‐enhancing effects comparable to those of transcranial direct current stimulation (tDCS) [15, 16], the present study adopted the Montreal Cognitive Assessment (MoCA) as the primary outcome measure, informed by the experimental results of a prior tDCS trial in PSCI patients, the EEG parameters were defined and analyzed as exploratory endpoints. The mean change in MoCA scores in the tACS group was 2.08 ± 1.11, compared to 1.08 ± 0.76 in the control group [17]. From these data, an effect size of 1.051 was calculated. To achieve a statistical power of 0.85 (1 − *β*) with a significance level (*α*) of 0.05, a minimum of 18 participants per group was required, totaling 36 participants. Accounting for an estimated 20% dropout rate, the final target sample size was set at 44 participants (22 per group).

### 2.4 Intervention Protocol

tACS was delivered using a battery‐driven current stimulator (Model: MBM‐IV; Jiangxi Huaheng Jingxing Medical Technology Co Ltd., China) through a pair of 4.5 × 4.5 cm saline‐soaked (0.9% NaCl) surface sponge electrodes. The stimulation targeted the left and right DLPFC, corresponding to the F3 and F4 electrode positions of the international 10/20 system. The alpha‐tACS group received 20‐min sessions of stimulation at 10 Hz and 2 mA peak intensity, administered once daily, 5 days per week, for 4 weeks. For the sham‐tACS group, the current was ramped up to 2 mA over the first 15 s and then ramped down to 0 mA, where it remained for the remainder of the 20‐min session.

Both groups received standardized cognitive rehabilitation based on the protocol by Li et al. [18], which comprised the following 30‐min daily sessions (5 sessions/week): orientation (calendars/cue cards), attention (visual tracking/letter cancellation), computation (graded arithmetic), memory (PQRST/picture recall/notepads), and executive function (daily scenario simulations).

### 2.5 Cognitive Assessment

The MoCA is a widely used screening tool with established sensitivity and specificity for cognitive impairment [19]. It evaluates multiple cognitive domains: visuospatial/executive function, naming, attention, language, abstraction, memory, and orientation. MoCA scores range from 0 to 30, with higher scores reflecting better cognitive performance. In this study, the change in MoCA score from baseline to post‐intervention was defined as the primary outcome measure.

Cognitive function was assessed at baseline and immediately following the final intervention session using a battery of tests including the MoCA and the Trail Making Test Parts A and B (TMT‐A, TMT‐B).

The TMT‐A and TMT‐B were administered to measure processing speed, attention, and executive function [20]. As a language‐independent cognitive assessment tool, the TMT effectively evaluates the functional status of these cognitive domains. The time taken to complete TMT‐A and TMT‐B represents the most commonly used metric for this test [21]. When used in conjunction with the Montreal Cognitive Assessment (MoCA), the TMT enables a more comprehensive observation of attention and executive function changes in patients with post‐stroke cognitive impairment (PSCI) before and after interventions, thereby providing valuable insights for clinical evaluation and rehabilitation planning.

### 2.6 Resting‐State EEG Data Acquisition

Resting‐state EEG data were recorded in a quiet, dimly lit room while participants were seated and instructed to remain awake and relaxed with their eyes closed. Data were acquired using a 19‐channel EEG system (Model: NVX 52; Manufacturer: Nanjing Zuoyou Brain Technology Co. Ltd.). The system's input impedance was > 100 MΩ. During recording, signals were band‐pass filtered between 0.5 and 30 Hz. Electrode impedances were maintained below 5 kΩ throughout the experiment.

Scalp electrodes were placed according to the International 10–20 System (Figure 1) at the following 19 sites: bilateral prefrontal region (Fp1, Fp2), frontal region (F3, F4, Fz), anterior temporal region (F7, F8), middle‐temporal region (T3, T4), posterior temporal region (T5, T6), central region (C3, C4, Cz), parietal region (P3, P4, Pz), and occipital region (O1, O2). The bilateral earlobes (A1, A2) served as the reference electrodes.

### 2.7 EEG Preprocessing and Alpha Power Spectral Density Analysis

EEG data were processed offline using the EEGLAB toolbox (v14.1.2) running on MATLAB R2017b (The MathWorks Inc.).

The preprocessing pipeline involved the following steps. First, the raw continuous data were band‐pass filtered from 0.5 to 45 Hz to remove signal drift and high‐frequency noise. The data were then downsampled from 1000 to 500 Hz to reduce computational load.

Following this, the continuous data were segmented into 2‐s epochs. Channels with poor signal quality (e.g., high impedance or signal loss) were identified by visual inspection and removed from further analysis.

Independent Component Analysis (ICA) was applied to the data to identify and remove stereotypical artifacts, including eye blinks, saccades, and muscle activity. After artifactual components were removed, the data underwent a final visual inspection to exclude any remaining epochs contaminated by noise or drift.

For the clean data, power spectral density (PSD) was computed for each channel and epoch using Welch's method (2‐s Hanning window, 50% overlap). The analysis focused specifically on the absolute power within the alpha frequency band (8–13 Hz).

### 2.8 EEG Analysis in DAR and DTABR

Following the PSD computation, the absolute power for each channel was calculated by integrating the PSD values within five standard frequency bands: Delta (0.5–4 Hz), Theta (4–8 Hz), Alpha (8–13 Hz), Beta 1 (13–20 Hz), and Beta 2 (20–30 Hz). Relative power for each band was then computed by dividing the absolute power of that band by the total power across the 0.5–30 Hz range. These relative power values were averaged across all 19 channels to yield a global mean relative power for each frequency band. Finally, two key power ratios were calculated: (1) Delta/Alpha Ratio (DAR): the ratio of global relative Delta power to global relative Alpha power. (2) DTABR: (Delta + Theta)/(Alpha + Beta) Ratio.

### 2.9 Statistical Analysis

All statistical analyses were performed using SPSS version 26.0 (IBM Corp., USA). Categorical variables were presented as frequencies or percentages and compared between groups using the chi‐square test or Fisher's exact test as appropriate. Continuous variables were expressed as mean ± standard deviation if normally distributed, and inter‐group comparisons were conducted using independent samples *t*‐tests, whereas paired samples *t*‐tests were used for within‐group comparisons.

For data that did not meet the assumption of normality, values were presented as median (interquartile range), and Mann–Whitney *U* tests were applied for inter‐group comparisons, whereas Wilcoxon signed‐rank tests were used for within‐group comparisons. Statistical significance was defined as *p* < 0.05. To mitigate the risk of Type I errors associated with multiple comparisons across the analyzed EEG metrics, a false discovery rate (FDR) correction was applied.

## 3 Results

### 3.1 Demographic and Clinical Characteristics

There were no significant differences between the two groups regarding age, gender, education level, disease duration, lesion side, lesion location, smoking, diabetes, and hypertension (all *p* > 0.05), see Table 1.

**TABLE 1:** Baseline demographic and clinical characteristics of patients.

Item	Alpha‐tACS group (*n* = 20)	Sham‐tACS group (*n* = 20)	*t*/*Z*/*x* ^2^	Cohen's *d*	*p*
Age	61.90 ± 8.31	61.80 ± 10.13	0.034	9.267	0.973
Gender (male/female)	11/9	12/8	0.102	—	0.999
Education level (years)	7.60 ± 3.66	7.60 ± 3.65	0.001	3.655	0.999
Lesion side (left/right)	7/13	8/12	0.107	—	0.999
Lesion location (cortical/subcortical)	3/17	3/17	0.001	—	0.999
Disease duration (days)	121.00 (99.00, 155.50)	110.50 (98.25, 162.75)	−0.298	32.277	0.779
Smoking (yes/no)	2/18	4/16	0.784	—	0.661
Diabetes (yes/no)	10/10	5/15	2.667	—	0.191
Hypertension (yes/no)	18/2	18/2	0.001	—	0.999

### 3.2 Changes in Cognitive Function Following Intervention

Prior to the intervention (T0), there were no significant differences between the alpha‐tACS and sham‐tACS groups in MoCA scores and Trail Making Test (TMT‐A and TMT‐B) completion times (*p* > 0.05).

After 4 weeks of intervention (T1), the MoCA score was significantly higher in the alpha‐tACS group than in the sham‐tACS group (*p* < 0.05). Additionally, TMT‐A and TMT‐B completion times were significantly shorter in the alpha‐tACS group compared to the sham‐tACS group (*p* < 0.05).

After 4 weeks of intervention (T1–T0), the difference in MoCA score was significantly greater in the alpha‐tACS group than in the sham‐tACS group (*p* < 0.05). After 4 weeks of intervention, the reduction in TMT‐A completion times in the alpha‐tACS group was significantly greater than that in the sham‐tACS group (*p* < 0.05). However, no significant difference was observed in the TMT‐B completion times between the two groups (*p* > 0.05), see Table 2.

**TABLE 2:** Comparison of neuropsychological test scores between groups before and after intervention.

Item	Alpha‐tACS group (*n* = 20)			Sham‐tACS group (*n* = 20)			T1‐0_Alpha group_ vs. T1‐0_Sham group_			T1_Alpha group_ vs. T1_Sham group_		
	T0	T1	T1–T0	T0	T1	T1–T0	*t*/*Z*	Cohen's *d*	*p*	*t*/*Z*	Cohen's *d*	*p*
MoCA score	15.70 ± 4.79	21.15 ± 3.82	5.45 ± 2.21	16.75 ± 5.29	17.60 ± 4.52	0.85 ± 1.87	7.100	2.049	**< 0.001**	2.687	4.178	**0.011**
TMT‐A time	168.60 ± 80.10	137.20 ± 60.95	−31.40 ± 31.45	184.75 ± 75.32	184.45 ± 78.03	−0.30 ± 20.36	−3.712	26.491	**0.001**	−2.134	70.012	**0.039**
TMT‐B time	300.00 (242.00, 300.00)	251.00 (199.00, 300.00)	−17.50 (−52.25, 0.01)	300.00 (298.50, 300.00)	300.00 (279.25, 300.00)	0.01 (−13.50, 0.01)	−1.802	31.430	0.073	−1.988	42.724	**0.048**

*Note:* Bold values indicate statistical significance (*p* < 0.05).

### 3.3 Changes in Alpha Power Spectrum Density Following tACS Intervention

At baseline (T0), there were no significant differences in alpha PSD at any EEG electrodes between the alpha‐tACS and sham‐tACS groups (*p* > 0.05).

After 4 weeks of intervention (T1), PSD at electrodes (FP1, FP2, F3, F4, C3, C4, P3, O1, O2, F7, F8, T5, T6, Fz, and Cz) was significantly higher in the alpha‐tACS group than in the sham‐tACS group (*p* < 0.05). Following FDR correction, the statistical outcomes remained largely consistent with the uncorrected results, with the exception of the P3 electrode, which no longer retained significance. In contrast, no statistically significant difference was observed in PSD at P4, T3, T4, and Pz electrodes between the two groups (*p*_FDR_ > 0.05).

After 4 weeks of intervention (T1–T0), the change in alpha PSD was significantly greater in the alpha‐tACS group than in the sham‐tACS group across all electrodes (*p*_FDR_ < 0.05), see Table 3. Furthermore, topological analysis of the EEG data revealed that the P3 electrode exhibited the most significant increase in PSD following the tACS intervention, see Figure 3.

**TABLE 3:** Differences in alpha power spectrum density and relative power ratios between groups before and after intervention.

Item	Alpha‐tACS group (*n* = 20)			Sham‐tACS group (*n* = 20)			T1‐0_Alpha group_ vs. T1‐0_Sham group_				T1_Alpha group_ vs. T1_Sham group_			
	T0	T1	T1–T0	T0	T1	T1–T0	*Z*	Cohen's *d*	*p*_uncorrect_	*p*_FDR_	*Z*	Cohen's *d*	*p*_uncorrect_	*p*_FDR_
*Alpha power spectrum density*														
FP1	6.40 (3.80, 13.05)	9.90 (5.93, 15.28)	1.60 (−0.45, 5.85)	4.25 (2.33, 6.80)	3.70 (2.75, 5.88)	−0.30 (−1.63, 1.00)	−2.354	5.511	**0.018**	**0.021**	−3.193	6.055	**0.001**	**0.001**
FP2	6.80 (4.15, 14.65)	10.70 (6.30, 15.75)	1.65 (−1.28, 6.10)	4.65 (3.03, 7.98)	3.80 (2.53, 6.20)	−0.75 (−2.55, 0.43)	−2.381	6.279	**0.016**	**0.021**	−3.071	6.812	**0.002**	**0.001**
F3	8.75 (5.48, 17.25)	11.90 (8.45, 24.50)	2.40 (0.80, 8.10)	7.30 (4.05, 10.18)	5.70 (4.00, 9.00)	−1.35 (−3.45, 1.23)	−3.071	7.198	**0.002**	**0.012**	−2.976	9.053	**0.002**	**0.001**
F4	9.95 (5.85, 21.15)	12.70 (8.85, 22.78)	2.75 (−0.38, 8.05)	7.10 (3.70, 11.38)	5.70 (3.70, 12.05)	−1.15 (−3.17, 0.68)	−2.787	9.670	**0.005**	**0.012**	−2.746	11.090	**0.005**	**0.014**
C3	8.20 (5.30, 26.25)	12.40 (7.43, 24.25)	3.15 (−1.83, 5.88)	8.35 (5.25, 11.58)	7.50 (4.73, 9.78)	−0.50 (−4.30, 0.78)	−2.137	7.672	**0.031**	**0.033**	−2.448	10.377	**0.013**	**0.021**
C4	9.00 (5.25, 24.00)	10.45 (7.78, 28.08)	2.95 (−1.30, 6.15)	8.25 (3.65, 14.45)	6.35 (4.23, 16.68)	−0.80 (−4.28, 1.03)	−2.326	9.982	**0.019**	**0.021**	−2.178	13.755	**0.029**	**0.039**
P3	15.77 ± 14.85	23.99 ± 19.58	8.22 ± 10.52	11.68 ± 8.72	10.92 ± 8.00	−0.76 ± 5.64	3.364	8.437	**0.002**	**0.012**	−2.002	14.956	**0.046**	0.058
P4	14.10 (4.85, 20.25)	15.50 (5.20, 34.90)	4.80 (0.70, 15.83)	9.70 (3.40, 15.83)	7.50 (3.55, 18.50)	−0.45 (−4.90, 2.05)	−2.840	9.78	**0.004**	**0.012**	−1.921	13.201	0.055	0.065
O1	16.10 ± 13.35	24.90 ± 19.53	8.81 ± 10.52	11.02 ± 8.51	9.28 ± 7.55	−1.75 ± 5.64	2.859	11.671	**0.009**	**0.016**	−2.895	14.809	**0.003**	**0.011**
O2	10.30 (4.93, 14.80)	18.40 (8.80, 31.68)	3.80 (−0.05, 16.65)	6.95 (2.83, 17.93)	4.65 (2.40, 18.60)	−1.70 (−4.35, 1.73)	−3.043	11.637	**0.002**	**0.012**	−2.530	10.645	**0.011**	**0.019**
F7	4.75 (2.83, 9.98)	7.10 (5.00, 11.08)	1.15 (−0.38, 3.85)	3.55 (2.05, 6.58)	3.10 (2.50, 5.63)	−0.55 (−1.40, 1.20)	−2.720	3.898	**0.005**	**0.012**	−2.707	4.329	**0.006**	**0.014**
F8	4.80 (2.95, 12.18)	7.05 (4.43, 10.28)	1.10 (−0.43, 4.25)	3.90 (2.23, 7.55)	2.80 (1.83, 7.03)	−0.85 (−1.95, 0.10)	−2.680	4.740	**0.007**	**0.015**	−2.652	5.483	**0.007**	**0.015**
T3	3.50 (2.30, 5.78)	5.20 (3.45, 8.38)	1.10 (0.35, 2.55)	3.10 (2.53, 6.18)	3.15 (2.00, 5.45)	−0.55 (−1.18, 0.83)	−2.841	3.219	**0.004**	**0.012**	−1.868	3.359	0.062	0.069
T4	4.40 ± 3.18	5.63 ± 4.09	1.23 ± 2.84	4.19 ± 3.00	3.90 ± 2.88	−0.30 ± 1.69	2.058	2.335	**0.048**	**0.048**	−1.543	3.536	0.126	0.126
T5	13.10 ± 13.21	17.38 ± 12.33	4.29 ± 8.34	9.46 ± 7.11	7.49 ± 6.09	−1.97 ± 5.84	2.746	7.197	**0.010**	**0.016**	−2.801	9.723	**0.004**	**0.013**
T6	9.90 (5.00, 16.85)	15.40 (6.85, 43.52)	5.20 (−0.58, 12.40)	7.00 (2.65, 15.65)	5.00 (2.85, 14.93)	−0.70 (−4.08, 2.48)	−2.922	9.349	**0.003**	**0.012**	−2.191	10.706	**0.028**	**0.039**
Fz	9.75 (7.40, 23.98)	14.50 (10.35, 27.60)	3.45 (−1.10, 11.23)	8.20 (4.78, 12.90)	6.45 (4.53, 11.85)	−0.55 (−3.20, 1.45)	−2.584	10.486	**0.009**	**0.016**	−2.989	12.627	**0.002**	**0.001**
Cz	13.55 (6.28, 26.53)	15.75 (9.88, 29.90)	3.75 (−0.18, 6.63)	10.25 (4.70, 17.20)	7.55 (6.05, 16.25)	−1.20 (−4.42, 1.93)	−2.530	11.673	**0.011**	**0.016**	−2.516	16.155	**0.011**	**0.019**
Pz	15.35 (5.28, 27.58)	18.40 (7.75, 41.18)	3.80 (−0.08, 13.15)	11.05 (4.45, 25.55)	8.70 (6.55, 19.45)	−0.35 (−6.40, 2.48)	−2.340	12.605	**0.018**	**0.021**	−1.722	17.041	0.077	0.081
*Relative power ratios*														
DAR global	1.36 (0.77, 2.96)	0.66 (0.41, 1.55)	−0.45 (−1.54, −0.33)	0.67 (0.47, 3.33)	0.67 (0.49, 2.77)	0.50 (−0.13, 0.35)	−4.127	1.623	**< 0.001**	**< 0.001**	−0.649	1.594	0.525	0.520
DTABR global	5.43 (0.98, 2.36)	1.05 (0.55, 1.66)	−0.57 (−1.20, −0.26)	1.03 (0.53, 3.34)	0.92 (0.65, 3.20)	0.05 (−0.36, 0.23)	−3.801	1.226	**< 0.001**	**< 0.001**	−0.730	1.691	0.474	0.520

*Note:* Bold values indicate statistical significance (*p* < 0.05).

### 3.4 Changes in Relative Power Ratios (DAR and DTABR) Following tACS Intervention

At baseline (T0), there were no significant differences between the alpha‐tACS and sham‐tACS groups in Delta/Alpha Ratio (DAR) and Delta + Theta/Alpha + Beta Ratio (DTABR) in the whole brain (*p*_FDR_ > 0.05).

After 4 weeks of intervention (T1), no statistically significant difference was observed in DAR and DTABR compared to the sham‐tACS group (*p*_FDR_ > 0.05).

After 4 weeks of intervention (T1–T0), the alpha‐tACS group had significantly greater reductions in DAR and DTABR values in whole brain compared to the sham‐tACS group (*p*_FDR_ < 0.05), see Table 3.

### 3.5 The Correlation Results Between the Difference of Alpha Power Spectral Density and the Difference of the TMT‐A Scale

**FIGURE 2:**
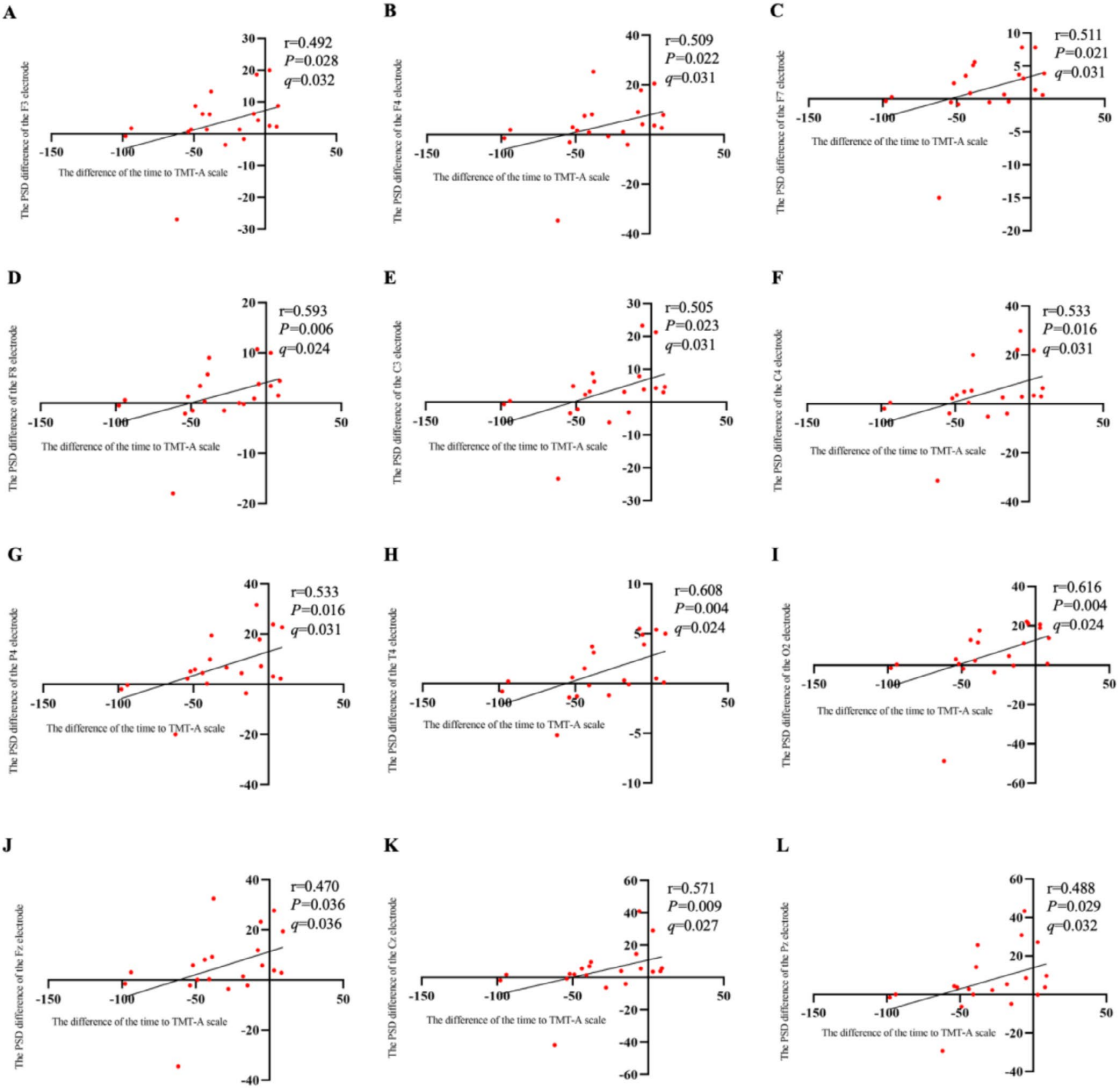
The correlation between the difference in alpha PSD and the difference in the TMT‐A scale. (A) The correlation between the difference in TMT‐A scores and the difference in PSD at the F3 electrode in the alpha‐tACS group. (B) The correlation between the difference in TMT‐A scores and the difference in PSD at the F4 electrode in the alpha‐tACS group. (C) The correlation between the difference in TMT‐A scores and the difference in PSD at the F7 electrode in the alpha‐tACS group. (D) The correlation between the difference in TMT‐A scores and the difference in PSD at the F8 electrode in the alpha‐tACS group. (E) The correlation between the difference in TMT‐A scores and the difference in PSD at the C3 electrode in the alpha‐tACS group. (F) The correlation between the difference in TMT‐A scores and the difference in PSD at the C4 electrode in the alpha‐tACS group. (G) The correlation between the difference in TMT‐A scores and the difference in PSD at the P4 electrode in the alpha‐tACS group. (H) The correlation between the difference in TMT‐A scores and the difference in PSD at the T4 electrode in the alpha‐tACS group. (I) The correlation between the difference in TMT‐A scores and the difference in PSD at the O2 electrode in the alpha‐tACS group. (J) The correlation between the difference in TMT‐A scores and the difference in PSD at the Fz electrode in the alpha‐tACS group. (K) The correlation between the difference in TMT‐A scores and the difference in PSD at the Cz electrode in the alpha‐tACS group. (L) The correlation between the difference in TMT‐A scores and the difference in PSD at the Pz electrode in the alpha‐tACS group.

Correlation analysis revealed that within the alpha‐tACS group, changes in TMT‐A scores were significantly positively correlated with changes in alpha PSD at the F3 electrode (*r* = 0.492, *p*_FDR_ = 0.032), F4 electrode (*r* = 0.509, *p*_FDR_ = 0.031), F7 electrode (*r* = 0.511, *p*_FDR_ = 0.031), F8 electrode (*r* = 0.593, *p*_FDR_ = 0.024), C3 electrode (*r* = 0.505, *p*_FDR_ = 0.031), C4 electrode (*r* = 0.533, *p*_FDR_ = 0.031), P4 electrode (*r* = 0.533, *p*_FDR_ = 0.031), T4 electrode (*r* = 0.608, *p*_FDR_ = 0.024), O2 electrode (*r* = 0.616, *p*_FDR_ = 0.024), FZ electrode (*r* = 0.470, *p*_FDR_ = 0.036), CZ electrode (*r* = 0.571, *p*_FDR_ = 0.027), and PZ electrode (*r* = 0.488, *p*_FDR_ = 0.032), see Figure 2.

### 3.6 The Regression Analysis and ROC Analysis of the Difference in Alpha Power Spectral Density

**FIGURE 3:**
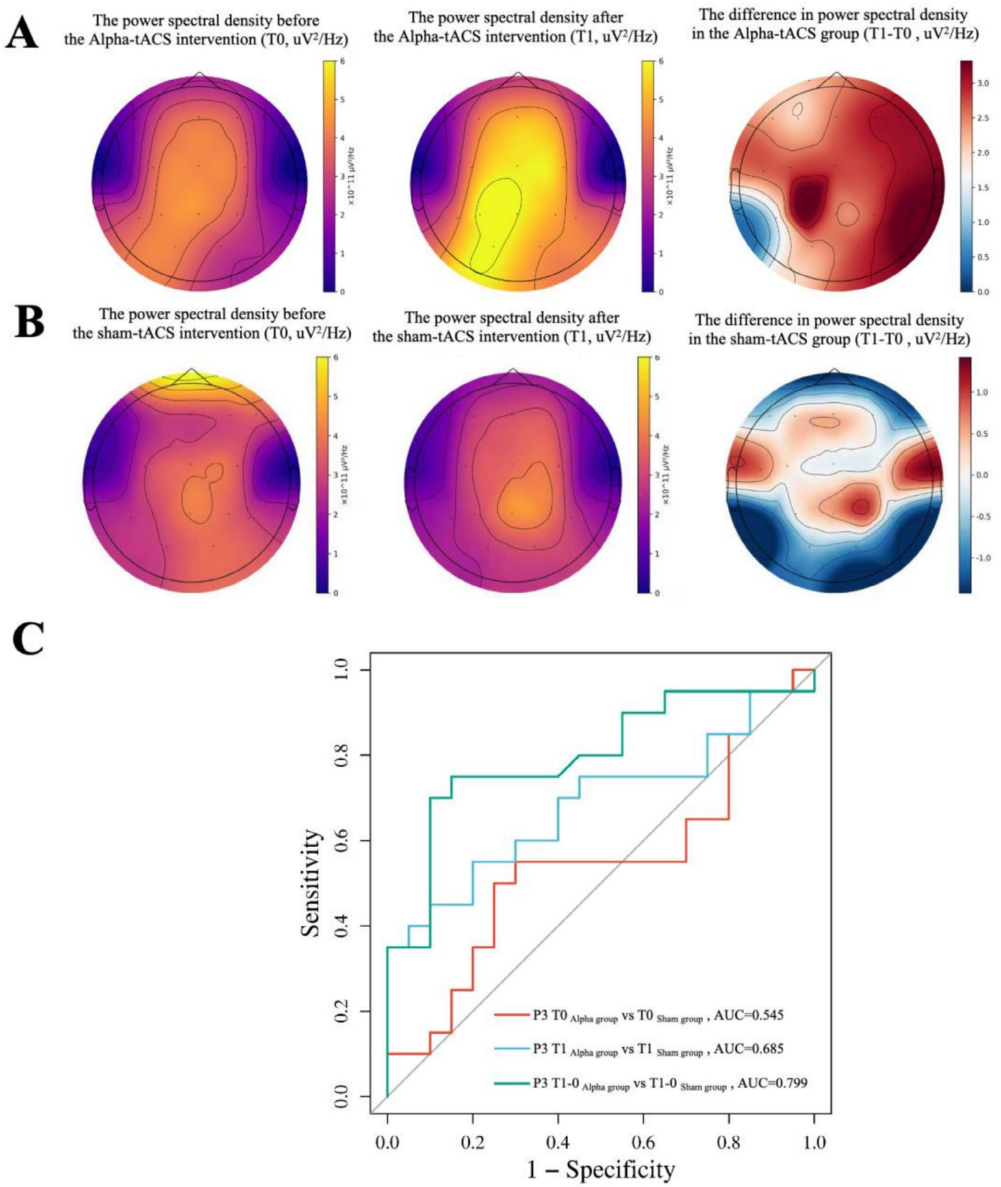
Electroencephalogram power topography and ROC analysis results. (A) EEG PSD topographic changes for alpha‐tACS group. The power spectral density before the alpha‐tACS intervention (T0, left), the power spectral density before the alpha‐tACS intervention (T1, middle), the difference in power spectral density in the alpha‐tACS group (T1–T0, right). (B) EEG PSD topographic changes for sham‐tACS group. The power spectral density before the sham‐tACS intervention (T0, left), the power spectral density before the sham‐tACS intervention (T1, middle), the difference in power spectral density in the sham‐tACS group (T1–T0, right). (C) ROC analysis results of the differences in P3 PSD among electrodes after tACS treatment.

The regression analysis using the stepwise method revealed that the PSD difference in P3 electrode was a significant predictor variable (*B* = −0.145, Wald *x* ^2^ = 6.668, *p* = 0.01, EXP(*B*) = 0.865, EXP(*B*) CI: 0.774–0.966). The ROC analysis results showed that the AUC at the P3 (T0 Alpha group vs. T0 Sham group) was 0.545. The AUC of P3 (T1 Alpha group vs. T1 Sham group) was 0.685. The AUC of P3 (T1‐0 Alpha group vs. T1‐0 Sham group) was 0.797, see Figure 3.

## 4 Discussion

This study investigated the effects of one‐month alpha‐tACS over the DLPFC on cognitive function and EEG activity in patients with PSCI. Compared to the sham‐tACS group, the alpha‐tACS group demonstrated significantly greater improvements in overall MoCA scores and TMT A completion times. PSD analysis revealed that alpha PSD at frontal, parietal, and occipital electrodes increased significantly after tACS intervention, relative to both baseline and the sham‐tACS group.

PSCI is a common sequela of stroke, typically characterized by deficits in attention, executive function, and memory. These cognitive deficits, which are observed in both ischemic and hemorrhagic stroke patients [22], can significantly hinder the rehabilitation process. Previous studies have reported varying degrees of spontaneous cognitive recovery within 3–6 months post‐stroke, although deficits in areas such as language tend to persist with limited improvement [23]. In the present study, alpha‐tACS notably improved the executive function of patients with PSCI.

These findings support the notion that alpha‐tACS may enhance cognitive recovery in PSCI, particularly in the domains of executive function and memory. This aligns with prior evidence demonstrating the cognitive‐enhancing effects of alpha‐band stimulation in various populations. For instance, He et al. [24] reported that applying 10 Hz tACS to the occipital cortex significantly improved visual perceptual learning—a key domain of cognitive function. Similarly, another study showed that 10 Hz tACS over the right DLPFC enhanced executive vigilance during a sustained attention‐to‐response task, likely by facilitating cognitive control through DLPFC activation [25]. In a clinical context, Alberto Benussi et al. applied alpha‐tACS in patients with dementia with Lewy bodies and found significant improvements in visuospatial‐executive performance, whereas language functions remained unaffected—echoing the findings of our study. Furthermore, their study also reported increased occipital alpha power following stimulation, which parallels our observed enhancement in parietal and occipital alpha activity [26]. These converging results suggest that alpha‐tACS may exert its cognitive benefits by strengthening intrinsic alpha oscillatory activity within brain networks involved in executive control and attention.

Previous research has demonstrated that alpha‐tACS can exert long‐term effects on endogenous alpha rhythms [8, 27], making it a promising non‐invasive intervention for alpha‐dependent neurological conditions such as post‐stroke disorders of consciousness and neuropsychiatric diseases associated with cognitive impairment [28, 29, 30]. Indeed, the enhancement of alpha power is often considered a biomarker for improved cognitive function, especially in domains such as attention and executive control [26, 31], a principle that aligns with the findings of the current study. The generation of alpha oscillations is believed to involve thalamocortical circuits [32, 33], with prominent activity in parietal‐occipital cortical regions that integrate visual and cognitive processing [34]. Stroke‐induced damage to these circuits has been implicated in the reduction of alpha activity in affected individuals [35, 36]. In this study, we observed a significant increase in alpha power at parieto‐occipital electrodes following 1 month of alpha‐tACS in PSCI patients, suggesting a possible mechanism by which alpha entrainment facilitates cognitive recovery.

Beyond absolute power, EEG power ratios are emerging as objective biomarkers for neurological disorders, with notable utility in assessing and monitoring PSCI. Elevated DAR in the frontal regions of stroke patients has been significantly associated with attentional deficits and global cognitive decline [37]. A recent meta‐analysis further demonstrated that DAR and DTABR have larger estimated effect sizes and correlate strongly with clinical outcomes such as the NIHSS and mRS scores [38]. Previous research has shown that patients within 3 months post‐ischemic stroke exhibit significantly elevated DTABR values across the frontal, central, temporal, and occipital cortices, which are negatively correlated with MoCA scores, suggesting DTABR as a dynamic marker for post‐stroke cognitive function [39]. In our study, a significant reduction in DAR was observed following alpha‐tACS, which corresponds with the observed improvements in executive and global cognitive function. Our findings indicate that alpha‐tACS effectively enhanced alpha power in the parietal and occipital regions while reducing the proportion of slow‐wave (delta and theta) activity, a shift reflected in the improvements in both DAR and DTABR. This modulation of abnormal slow‐fast wave balance underlies the observed cognitive improvements and provides mechanistic support for the application of alpha‐tACS in PSCI rehabilitation.

The DLPFC plays a critical role in the regulation of executive function and attention and is one of the most commonly targeted regions in non‐invasive neuromodulation treatments for PSCI [40, 41]. As a key hub within the frontoparietal control network, the DLPFC maintains strong functional connectivity with parietal and occipital cortices, supporting high‐level cognitive processes such as working memory, attentional control, and cognitive flexibility [42]. In this context, alpha‐tACS targeting the DLPFC may enhance alpha oscillatory activity within this region, thereby reinforcing functional coupling between the DLPFC and posterior brain regions. This strengthened connectivity could underlie the observed improvements in attention and executive function following stimulation. Stroke‐induced cognitive impairments are often accompanied by disruptions in large‐scale brain networks; thus, enhancing oscillatory synchronization through tACS may facilitate the restoration and reorganization of these networks [43]. The increased alpha power observed over the parietal and occipital regions may therefore reflect improved functional integrity within this network, contributing to the executive and cognitive recovery observed in PSCI patients.

The present study identified the change in PSD at the P3 electrode as a key indicator of the tACS intervention's effect. Stepwise regression analysis confirmed that the PSD difference at P3 was a significant predictor of the outcome. This finding is further corroborated by the ROC analysis, which demonstrated a progressive increase in the model's discriminative power. Specifically, while the baseline (T0) PSD at P3 yielded an AUC of 0.545, indicating initial group similarity, this value increased moderately post‐intervention (to 0.685 at T1). Most importantly, the change in PSD from baseline (T1–T0) proved to be the strongest classifier, achieving a good discriminative ability with an AUC of 0.797. This suggests that the tACS‐induced change in neural activity at the left parietal cortex (represented by P3), rather than its absolute state, serves as a robust biomarker for differentiating the effects of active stimulation from sham stimulation.

Several limitations should be considered. First, the modest single‐center sample focused on subcortical PSCI may affect generalizability; larger multicenter trials including diverse stroke types are needed. Second, the lack of follow‐up after the 4‐week intervention limits conclusions about effect durability; future studies should incorporate longer‐term assessments. Third, the fixed stimulation parameters preclude optimization; comparative studies of different protocols are warranted to refine treatment efficacy. Besides, the neurophysiological outcomes, including alpha power and spectral ratio indices, were prespecified as exploratory endpoints aimed at elucidating potential mechanisms. Consequently, the study was not specifically powered for these EEG metrics, and the findings should be interpreted as hypothesis generating. Future large scale studies with sample sizes determined by reliable electrophysiological effect size estimates are needed to confirm these preliminary observations.

## 5 Conclusion

This study demonstrates that alpha‐tACS applied to the DLPFC significantly improves executive function in patients with PSCI. EEG analysis revealed that there was a significant increase in alpha power in the frontal, parietal, and occipital regions following stimulation, suggesting that the executive improvements may be mediated by increasing alpha oscillatory activity.

## Funding

This work was supported by Fujian Provincial Health Commission Medical Innovation Project (2024CXA048), Rehabilitation technology innovation center by joint collaboration of ministry of education and Fujian province, Fujian University of traditional Chinese Medicine (X2022004), Fujian Province University Industry‐University Cooperation Project (2025Y4008).

## Ethics Statement

The study was conducted in accordance with the Declaration of Helsinki (2013 revision) and the protocol was approved by the Ethics Committee of the Rehabilitation Hospital of Fujian University of Traditional Chinese Medicine.

## Conflicts of Interest

The authors declare no conflicts of interest.

## Data Availability

The data that support the findings of this study are available from the corresponding author upon reasonable request.
